# Learning linear transformations between counting-based and prediction-based word embeddings

**DOI:** 10.1371/journal.pone.0184544

**Published:** 2017-09-19

**Authors:** Danushka Bollegala, Kohei Hayashi, Ken-ichi Kawarabayashi

**Affiliations:** 1 Department of Computer Science, University of Liverpool, Liverpool, United Kingdom; 2 National Institute of Advanced Industrial Science and Technology, Tokyo, Japan; 3 National Institute of Informatics, Tokyo, Japan; 4 Kawarabayashi ERATO Large Graph Project, Tokyo, Japan; Universitat Rovira i Virgili, SPAIN

## Abstract

Despite the growing interest in prediction-based word embedding learning methods, it remains unclear as to how the vector spaces learnt by the *prediction-based* methods differ from that of the *counting-based* methods, or whether one can be transformed into the other. To study the relationship between counting-based and prediction-based embeddings, we propose a method for learning a linear transformation between two given sets of word embeddings. Our proposal contributes to the word embedding learning research in three ways: (a) we propose an efficient method to learn a linear transformation between two sets of word embeddings, (b) using the transformation learnt in (a), we empirically show that it is possible to predict distributed word embeddings for novel unseen words, and (c) empirically it is possible to linearly transform counting-based embeddings to prediction-based embeddings, for frequent words, different POS categories, and varying degrees of ambiguities.

## 1 Introduction

Representing the meaning of a word is a fundamental task in Natural Language Processing (NLP). Two main approaches for computing word embeddings can be identified in the literature: *counting-based* approaches, and *prediction-based* approaches [1].

Counting-based methods represent a target word by the words that co-occur with that target word in various contexts using some co-occurrence measure [2]. Following the distributional hypothesis that states the meaning of a word can be represented using the words that co-occur with that word in different contexts, for example, the words that co-occur with the word *cat* such as *pet food*, *dog*, *cute* etc. can be used to represent the meaning of *cat*. Various association measures such as the pointwise mutual information (PMI), *χ*^2^ measure, log likelihood ratio (LLR) have been proposed in the literature for measuring the strength of the co-occurres between two words. Any word in the vocabulary (i.e. the set consisting of all words in a language) can appear in a co-occurring context. Consequently, under counting-based approaches, a word is represented in a high dimensional (in practice dimensionality greater than 10^5^ are common) vector space. However, among all the words in the vocabulary only a handful of words will be co-occurring with any given word. Therefore, counting-based embeddings tend to be highly sparse. This becomes problematic when applying counting-based word embeddings as features for representing words in downstream NLP applications such as similarity measurement, or sentiment classification because of feature sparseness. To overcome these disfluencies associated with counting-based word embeddings dimensionality reduction methods such as Singular Value Decomposition (SVD) are often used as a post-processing step.

Prediction-based word embedding learning methods [3–5] on the other hand update fixed dimensional word vectors (possibly randomly initialized) such that we can accurately predict the words that appear in a target word in a given context. Prediction-based methods have reported impressive performances across a wide range of NLP tasks such as sentiment classification [6], named entity recognition [7], semantic role labeling [8], and machine translation [9]. Prediction-based word embedding learning methods produce lower dimensional (ca. 100 − 1000 dimensions are common) and dense word representations. The vector spaces spanned by the prediction-based embeddings are known to demonstrate a certain level of linear structure where subtraction of word embeddings result in vectors that represent the semantic relationships between two words. For example, embedding produced by *v*(king) − *v*(man) + *v*(woman) is shown to be similar to *v*(queen), where we use the notation *v*(*x*) to denote the embedding of the word *x*. Unfortunately, the lower-dimensional dense embeddings produced by the prediction-based methods are difficult to interpret compared to the high-dimensional and sparse representations produced by the counting-based methods where each dimension can be explicitly identified with a context word.

Despite the success stories of prediction-based embeddings, we understand very little about how they differ from their counting-based counterparts. Levy et al. [10] empirically showed that the differences between the two types of embeddings can be mainly attributable to the differences in hyperparameter settings and pre-processing steps. Intuitively, given that both types of embeddings are learnt from the same source of data, we would expect some relationship between prediction-based and counting-based embeddings. More specifically, because of the high-dimensionality of the counting-based embeddings, they could potentially preserve the information captured by the prediction-based embeddings.

But how can we investigate the relationship between these two types of embeddings? Because the two types of embeddings have different dimensionalities, a direct comparison is impossible. However, if the counting-based methods truly capture the same information as the prediction-based methods, then we must be able to recover prediction-based embeddings from the counting-based embeddings. In other words, there must exist a *projection* from the high-dimensional, counting-based word-embedding space to the low-dimensional, prediction-based word-embedding space.

In this paper, we investigate such a projection with the simplest possible setting—*linearity*. We propose a method that learns the optimal linear transformation between two given sets of embeddings in a supervised way (i.e., the error between the target and the transformed embeddings is minimized). Because of the cheap computational cost, the linearity assumption allows us to compare a large number (ca. 400*k*) of word embeddings to obtain statistically reliable results. Moreover, if word embeddings can be converted using linear projections, then it provides empirical evidence to the fact that the vector spaces learnt by the prediction-based word embedding learning methods are linear in structure.

Our experiments bring a few surprising results (Sections 4.1 and 4.2). First, when a transformation is sufficiently optimized in terms of the training error, the projected embeddings achieve the same performance as the prediction-based embeddings in similarity measurement tasks. Moreover, it is possible to learn accurate linear transformations for frequent words, irrespective POS category. These results plausibly support our hypothesis: the counting-based methods inclusively contain the same information as the prediction-based methods.

Aside from the above-mentioned findings, the linear transformation method itself is useful. One disadvantage of prediction-based methods is that we must retrain *all* word embeddings when we want to learn the word embeddings for novel words that were later added to the corpus. On the other hand, it is relatively easier to create counting-based word embeddings for a novel word because we require contexts in which only that novel word occurs. In Section 4.2, we show that the word embeddings predicted using the linear transformations learnt by the proposed method correctly predict the semantic similarity and word analogies in several benchmark datasets.

## 2 Related work

Word embedding learning has received a renewed interest lately due to the impressive performances obtained by the prediction-based word embedding learning methods in a wide range of NLP applications such as sentiment classification [6, 11, 12], named entity recognition [13, 14], word sense disambiguation [15, 16], relation extraction [17, 18], semantic role labeling [8], and machine translation [9].

Mikolov et al. [4] proposed prediction-based two word embedding learning methods: skip-gram with negative sampling (SGNS), and the continuous bag-of-words model (CBOW). In SGNS, the word embedding for a target word is learnt such that we can correctly predict each of the context words in a given co-occurrence context such as a sentence or a pre-defined fixed window of tokens. On the other hand, in CBOW, all words in a given context are used to jointly predict a particular target word. A log bi-linear model is used to approximate the probability of two words co-occurring in a given context. The word embeddings are learnt such that the likelihood of the predictions is maximized over the entire corpus.

Pennington et al. [3] proposed *global vector prediction* (GloVe), a prediction-based word embedding learning method, where word embeddings of the target and context words are learnt such that they can accurately predict the logarithm of the co-occurrence count between those two words. Unlike, SGNS or CBOW, GloVe considers the global co-occurrences of two words computed over the entire corpus.

However, our goal in this work is *not* to propose a new word embedding learning method, but to learn a linear transformation between two given sets of word embeddings. In Section 3.2, we describe several prediction-based word embedding learning methods such as the global vector representation (GloVe) [3], continuous bag-of-words model (CBOW), and skip-gram with negative sampling (SGNS) [19], which we use in our evaluations.

Levy et al. [10] empirically showed that the differences in performances obtained using counting-based and prediction-based embeddings can be largely attributable to the different hyperparameter settings and pre-processing steps. Moreover, some prediction-based word embedding learning methods such as GloVe and SGNS have been shown to factorize some form of a transformed co-occurrence matrix, similar to the ones used by the counting-based word embedding methods [20–22]. Such prior studies hint at a close relationship between the two approaches for learning word embeddings.

Faruqui et al. [23] created non-distributional word embeddings using attributes specific to words from a collection of manually created lexical resources. These word representations are high-dimensional and sparse. The dimensions of these word representations are interpretable because they correspond to various relations defined in the lexical resources. The linear transformation we learn between counting-based and prediction-based embeddings can be seen as an empirical method to interpret the implicit dimensions in the prediction-based embeddings using a linear combination of the explicit dimensions in the counting-based embeddings. We can use the non-distributional word representations created by Faruqui et al. [23] as one of the source embeddings in our proposed method.

Mitchell and Steedman [24] observed that word embeddings can be decomposed into semantic and syntactic components. They proposed a method for learning word embeddings that encode word-order and morphology that outperformed embeddings trained using CBOW, SGNS and GloVe. We believe the insights we obtain in this paper about the structure of the vector spaces learnt by the word embedding learning methods will be useful to further improve word embedding learning methods.

## 3 Method

As introduced in Section 1, two main approaches exist for learning word embeddings: counting- and prediction-based. Given two sets of vector embeddings defined over a common vocabulary, in Section 3.1, we propose a method that learns a *linear transformation* between the vector spaces spanned by the two sets of embeddings. Next, in Section 3.2, the learnt linear transformation is used to study the differences between several counting-based and prediction-based embeddings.

The reasons for limiting the transformations we consider to linear ones are two-fold. First, most prediction-based word embedding learning methods differ only in the way they optimize different loss functions measuring the accuracy of the prediction, and how they set the numerous hyperparameters [10]. Moreover, prediction-based embedding learning methods such as GloVe and SGNS can be seen as factorizing word co-occurrence matrices transformed by suitable operations such as the logarithm of the co-occurrence frequency or shifted positive pointwise mutual information (PPMI) [20, 21]. Therefore, it is reasonable to assume that linear relationships would hold between embeddings learnt by different methods at least for the majority of the words. More importantly, we can empirically evaluate the deviation from the learnt linear transformation for any given word, thereby obtaining useful insights as to how the existing embedding learning methods differ in practice.

Second, in contrast to learning linear transformations, learning multivariate non-linear relationships between large sets of vectors is computationally expensive [25]. Considering that we would like to conduct a large-scale study involving a large number of embeddings to obtain statistically meaningful comparisons, linear transformations are computationally attractive. We defer the study of efficient non-linear transformation learning methods to future work.

### 3.1 Learning linear transformations

Let us consider two word embedding learning methods, which we refer to as the source (S) and the target (T) embedding learning methods, for learning word embeddings for a vocabulary $V={\left\{w_{i}\right\}}_{i=1}^{n}$ consisting of *n* words. For a word *w*_*i*_, let us denote the embeddings learnt by S and T respectively by vectors $w_{i}^{\left(S\right)}\in {\mathbb{R}}^{d}$, and $w_{i}^{\left(T\right)}\in {\mathbb{R}}^{p}$ (*d* ≠ *p* in general). We arrange the embeddings learnt by S as rows to create a matrix **S** ∈ ℝ^*n* × *d*^. Likewise, the embeddings learnt by T are arranged as rows to create a matrix **T** ∈ ℝ^*n* × *p*^. Then, we propose a method to learn a linear transformation from S to T, described by a transformation matrix **C** ∈ ℝ^*d* × *p*^, which minimizes the transformation loss, *J*(**C**), given by Eq (1).

$$\begin{array}{c} J\left(C\right)=||SC-{T||}_{F}^{2}+{\lambda ||C||}_{F}^{2} \end{array}$$ (1)

Here, λ ∈ ℝ is the *l*_2_ regularization coefficient, and ${\left|\left|\text{A}\right|\right|}_{F}=\sqrt{\text{tr}\left(\text{A}{\;}^{⊤}\text{A}\right)}$ is the Frobenius norm of **A**.

Eq (1) defines a multivariate regularized least square problem [26] where, **C** can be seen as a linear projection from S to T. Although we described the transformation learning problem in Eq (1) as learning a projection from S to T, the inverse transformation can be learnt by simply swapping S and T in Eq (1).

Eq (1) can be written using matrix trace as follows:

$$J\left(C\right)=(\left(SC-T\right){\left(SC-T\right)}^{⊤})+\lambda \text{tr}\left(C{\;}^{⊤}\;C\right)$$ (2)

From Eq (2) we can compute the partial derivative of the loss w.r.t. **C** as follows:

$$\frac{\partial J}{\partial C}=2S^{⊤}SC-2S^{⊤}T+\text{2}\lambda C$$ (3)

By setting $\frac{\partial J}{\partial C}$ to zero we can compute **C** in closed-form as follows:

$$\text{C}={\left(S^{⊤}S+\lambda {\text{I}}_{d}\right)}^{-1}S^{⊤}T$$ (4)

Here, **I** *_d_* ∈ ℝ^*d* × *d*^ is a unit matrix.

For the counting-based embeddings, which are sparse and high-dimensional, **S**^⊤^ **S** results in a dense *n* × *n* matrix. Therefore, the inversion of a possibly dense matrix in the size of the vocabulary required by Eq (4), is computationally costly in practice, except for the smallest of corpora.

In practice, however, stable numerical solutions can be found efficiently by using an SGD algorithm. A key idea is that the objective function is decomposable as

$$\begin{array}{c} J=\sum_{i=1}^{n}{\tilde{J}}_{i},\;{\tilde{J}}_{i}=|\left|Cs_{i}-t_{i}\right|{|}_{F}^{2}+\frac{\lambda }{n}{\left|\left|C\right|\right|}_{F}^{2}, \end{array}$$ (5)

where **s** *_i_* and **t** *_i_* are the *i*-th row vector of **S** and **T**, respectively. By constructing the gradient of ${\tilde{J}}_{i}$ instead of *J*, we can update **C** in a stochastic way as follows:

$$\begin{array}{c} C^{\left(t+1\right)}=C^{\left(t\right)}-\eta^{\left(t\right)}\frac{\partial \tilde{J}}{\partial C} \end{array}$$ (6)

Here, the superscript *t* denotes the value at the *t*-th iteration, and *η*^(*t*)^ is the learning rate, scheduled using AdaGrad [27]. The stochastic gradient $\frac{\partial \tilde{J}}{\partial C}$ is written in a similar manner to the batch gradient Eq (3).

Once a linear transformation matrix **C** is learnt between a pair of source and target embedding methods, given the source embedding $w^{\left(S\right)}$ of a word *w*, we can predict its target embedding, ${\hat{w}}^{\left(T\right)}$ using Eq (7).

$$\begin{array}{c} {\hat{w}}^{\left(T\right)}=Cw^{\left(S\right)} \end{array}$$ (7)

### 3.2 Comparing word embeddings

The method proposed in Section 3.1 for learning a linear transformation between two given word embeddings S and T can be used to compare arbitrary embedding learning methods. Specifically, we can first create word embeddings using S and T for a common set of words, and use the method described in Section 3.1 to learn a linear transformation **C**. However, as representative cases, we focus on linear transformations between three counting-based word embedding methods (**RAW**, **LOG**, **PPMI**) and three popular prediction-based word embedding methods (**SGNS**, **CBOW**, and **GloVe**) as described next.

**Counting-based Embeddings**
RAW: We create distributional word representations by representing each target word *u*_*i*_ using the words *v*_*j*_ that co-occur with *u*_*i*_ within some contextual window in a corpus. The value of the *j*-th dimension of the embedding representing *u*_*i*_ is set to the total number of co-occurrences, *h*(*u*_*i*_,*v*_*j*_), between *u*_*i*_ and *v*_*j*_ in the entire corpus.
LOG: The value of the *j*-th dimension of this embedding representing *u*_*i*_ is set to log(*h*(*u*_*i*_,*v*_*j*_) + 1). Here, the +1 term prevents the logarithm from exploding when *h*(*u*_*i*_,*v*_*j*_) = 0. The logarithmic co-occurrence weighting has been found to be effective for down-weighting co-occurrences with high-frequency words [2].
PPMI: The value of the *j*-th dimension of this embedding representing *u*_*i*_ is set to to the positive pointwise mutual information (PPMI) between *u*_*i*_ and *v*_*j*_ computed by,
  $$\text{PPMI}(u_{i}v_{j})=\text{max}\left(0,\text{log}\left(\frac{p(u_{i},v_{j})}{p\left(u_{i}\right)p\left(v_{j}\right)}\right)\right).$$ (8)
  Here, the probabilities *p*(*u*_*i*_,*v*_*j*_), *p*(*u*_*i*_), *p*(*v*_*j*_) are estimated using corpus counts. If the occurrence of *u*_*i*_ subsumes the occurrence of *v*_*j*_ (i.e. *p*(*u*_*i*_,*v*_*j*_) = *p*(*u*_*i*_)), then *PPMI* simplifies to $\text{PPMI}(u_{i}v_{j})=\text{max}\left(0,\text{log}\left(\frac{1}{p\left(v_{j}\right)}\right)\right)$. Therefore, if the occurrence of *v*_*j*_ is rare (i.e. *p*(*v*_*j*_) ≈ 0), then its *PPMI* value with *u*_*i*_ becomes higher. This shows that *PPMI* has a tendency to overestimate rare co-occurrences, which can be problematic when co-occurrence counts are sparse.

**Prediction-based Embeddings**
SGNS: Skip-gram with negative sampling (SGNS) [19] learns target and context word embeddings by predicting the context words that co-occur with a target word in some context. The probability *p*(*v*_*j*_|*u*_*i*_) of observing the context word *v*_*j*_ in the proximity of a target word *u*_*i*_ is computed using the inner-product between the corresponding embeddings as given by Eq (9).
  $$\begin{array}{c} p(v_{j}|u_{i})=\frac{\text{exp}\left(u_{i}{\;}^{⊤}\;v_{j}\right)}{\sum_{j}\text{exp}\left(u_{i}{\;}^{⊤}\;v_{j}\right)} \end{array}$$ (9)
  The normalization term in Eq (9) requires a summation over all the context words, which is computationally costly. Alternatively, SGNS uses a negative sampling method based on noise contrastive estimation [28], where the log-likelihood over the entire corpus is maximized by comparing each target word with a randomly selected few context words that do not co-occur in a given context.
CBOW: In contrast to SGNS, the continuous bag-of-words model (CBOW) [4] predicts *all* context words *v*_*j*_ in a given context that co-occur with a target word *u*_*i*_. Similar to Eq (9), the joint conditional probability, *p*(*v*_1_,…,*v*_*j*_|*u*_*i*_), is computed using a log-bilinear function where the context word embeddings are concatenated to create a single context vector with which the inner-product of ***u**_i_* is computed.
GloVe: Unlike SGNS and CBOW which learn word embeddings by predicting the co-occurrences between target and context words within a specific local context, the global vector representation (GloVe) [3] method learns word embeddings by predicting the global co-occurrence counts between a target word *u*_*i*_, and a context word *v*_*j*_, obtained from the entire corpus. Specifically, GloVe learns word embeddings ***u**_i_*, ***v**_i_* by minimizing the squared loss taken over all pairs of target and context words as given by Eq (10).
  $$J\left({\left\{u_{i}\right\}}_{i=1}^{n},{\left\{v_{j}\right\}}_{j=1}^{n}\right)={\sum_{i,j}\left(u_{i}{\;}^{⊤}\;v_{j}-\text{log}h(u_{i}v_{j})\right)}^{2}$$ (10)

## 4 Experiments

Our proposed method learns a linear transformation between two pre-trained sets of word embeddings representing a common set of words. However, direct manual evaluation of linear transformations is infeasible due to the scale of the transformation matrix. Instead, we resort to a series of indirect extrinsic evaluation tasks as described next.

In Section 4.1, we evaluate the ability of the learnt linear transformation **C** to predict the target embedding $w^{\left(T\right)}$, of a word, *w*, given its source embedding $w^{\left(S\right)}$ using Eq (7). This experiment reveals (a) how well **C** fits to train word embeddings, thereby demonstrating the ability to linearly transform embeddings of different categories of words such as by frequency in a corpus, POS category, and polysemy, and (b) how well **C** can predict the target embedding for unseen test words.

In Section 4.2, we compare the level of semantic information retained during the transformation learning process by evaluating the predicted target embeddings using two standard evaluation tasks for word embeddings: semantic similarity measurement, and word-analogy detection.

### 4.1 Predicting embeddings for novel words

We use the ukWaC (http://wacky.sslmit.unibo.it/doku.php?id=corpora), a ca. 2 billion token corpus consisting of a Web crawl from the .uk domain. It has been used extensively in prior work on word embedding learning. We trained word embeddings for GloVe (http://nlp.stanford.edu/projects/glove/), CBOW, and SGNS (https://code.google.com/archive/p/word2vec/) using the original implementations. From ukWaC, we randomly select *n* = 434,804 words that occur at least 20 times as **train** words and select the corresponding prediction-based embeddings. We use all words that co-occur within a five-token window with the train words to create *d* = 434,826 dimensional counting-based embeddings. Word embeddings are randomly initialised sampling from a zero mean and unit variance Gaussian distribution. Negative sampling rate is set to 5 in SGNS and CBOW (i.e. 5 negative samples are selected per single occurrence of a target word). Due to space limitations, we report results with *p* = 300 dimensional embeddings for all three prediction-based embedding methods.

Although in theory it is possible to use the proposed method to learn transformations between two counting-based word embeddings, or two prediction-based word embeddings as well, our goal in this paper is to understand the differences between counting-based and prediction-based embeddings. Therefore, we set the source embedding S to each of the counting-based embeddings (**RAW**, **LOG**, **PPMI**) and target embeddings to each of the prediction-based embeddings (**SGNS**, **CBOW**, **GloVe**), and learn separate linear transformations **C** for each S-T pair.

To obtain **C**, we use Vowpal Wabbit (https://github.com/JohnLangford/vowpal_wabbit), a linear regression solver based on SGD. In this algorithm, we have two hyperparameters: regularization coefficient λ in Eq (1) and the number of learning passes *π* in SGD. We tuned these hyperparameters by grid search. Specifically, we randomly picked three hundred words for validation, and based on that we selected the best combination of λ and *π* from λ ∈ {10^−2^,10^−3^,…,10^−6^} and *π* ∈ {2^0^,2^1^,…,2^6^}. Selected hyperparameters are shown in Table 1. From Table 1 we see that λ is relatively insensitive to the source and target embeddings, whereas smaller *π* are suitable for **RAW** source embeddings.

**Table 1:** The hyperparameters used in the prediction tasks.

T (pred)	S (count)	λ	*π*
**SGNS**	**RAW**	10^−4^	2
	**LOG**	10^−4^	64
	**PPMI**	10^−4^	16
**CBOW**	**RAW**	10^−4^	16
	**LOG**	10^−5^	64
	**PPMI**	10^−4^	16
**GloVe**	**RAW**	10^−5^	1
	**LOG**	10^−5^	32
	**PPMI**	10^−2^	16

The testing error was then calculated against three hundred words selected as follows. We first compute the frequencies of words in the corpus and order the words in the descending order of their frequencies. Next, we select 100 words from high-frequency range (i.e. the words whose ranks were in 1–10,000) another 100 words from the medium-frequency range (i.e. the words whose ranks were in 10,001–20,000), and another 100 words from the low-frequency range (i.e. the words whose ranks were in 20,001–30,000). In particular, we ensure that the validation and test datasets do not include any words from the similarity and analogy benchmarks used in the experiments in Section 4.2.

To evaluate the accuracy of the predicted target embeddings using Eq (7) with a learnt transformation **C**, we compute the root mean square error (RMSE) between the predicted, ${\hat{w}}^{\left(T\right)}$, and target, $w^{\left(T\right)}$ embeddings over a set of words, V, as follows:

$$\begin{array}{c} \text{RMSE}=\sqrt{\frac{1}{\left|v\right|}\sum_{w\in v}\|{\hat{w}}^{\left(T\right)}-w^{\left(T\right)}{\|}_{2}^{2}} \end{array}$$ (11)

If the RMSE between the projected embedding of a word and its target embedding is small, then we can conclude that it is possible to linearly project from the source to the target embedding for that particular word. By repeating this process over a large set of words, and by computing the average RMSE for the entire set of words, we can quantitatively evaluate the accuracy of the learnt linear transformation.

In Table 2, we compare the proposed method (**Prop**) against a random projection baseline (**Rand**), where we project the *d*-dimensional source embeddings onto a *p*-dimensional space using a random projection matrix, **R** ∈ ℝ^*d* × *p*^, in which each element is randomly sampled from a standard (zero mean and unit variance) Gaussian distribution. From Table 2, we see that both train and test error values for the proposed method is smaller than that of the corresponding random projection. This result shows that the proposed linear transformation learning method outperforms the random projection baseline in both fitting the train word embeddings as well as predicting the test word embeddings.

**Table 2:** Train and test RMSE values when predicting target embeddings (SGNS, CBOW, GloVe) using different source embeddings (RAW, LOG, PPMI).

Method	S	SGNS		CBOW		GloVe	
		test	train	test	train	test	train
**Prop**	**RAW**	.200	.111	.151	.072	.332	.206
	**LOG**	.151	.095	.127	.060	.241	.178
	**PPMI**	.152	.090	.130	.065	.345	.218
**Rand**	**RAW**	.230	.147	.169	.096	.358	.228
	**LOG**	.230	.147	.168	.096	.359	.228
	**PPMI**	.231	.147	.169	.096	.358	.228

When we use the proposed method to learn linear transformations, among the three counting-based embedding methods **LOG** gives the smallest test error for all prediction-based target embedding methods, followed by **PPMI**, which has a tendency to over-estimate rare co-occurrences. In particular, **RAW** has the largest test error when predicting test word embeddings learnt using **SGNS** and **CBOW**. This result shows that some form of a co-occurrence weighting method is necessary with counting-based embeddings, if they are to be linearly transformed to prediction-based embeddings. Interestingly, **LOG** performs best with **GloVe**, which can be seen as factorizing a co-occurrence matrix containing the logarithms of the global co-occurrence counts [21]. As for **SGNS**, which is shown to be factorizing a matrix with shifted PPMI values [20], we do not see any significant differences between test errors reported for **LOG** and **PPMI**.

**LOG** as the co-occurrence weighting method for the counting-based word embedding method produces the lowest test prediction error with all of the prediction-based word embeddings. Both **SGNS** and **CBOW** are log bi-linear models. The logarithm of the co-occurrence probabilities estimated using those models are proportional to the inner-product between the corresponding word embeddings. By considering the log co-occurrences in the counting-based word embeddings, we can better approximate the linearities present in those prediction-based embedding spaces.

To study the prediction error for different POS categories, we classify each test word into one of the four POS categories, *noun*, *verb*, *adjective*, or *adverb*, according to the POS category assigned to the first-ranked sense of that word in the WordNet (https://wordnet.princeton.edu/). Next, we compute test error over the words classified to each of the four POS categories as shown in Table 3. Although there are slight variations in the prediction errors across different POS categories, an analysis of variance (ANOVA) test shows that the differences to be statistically insignificant. Therefore, we are unable to find any significant differences between the POS types.

**Table 3:** Testing RMSEs for different counting-based (count.) embeddings as the source, and prediction-based (pred.) as the target for different POS categories.

T (pred)	S (count)	Noun	Verb	Adj.	Advb.
**SGNS**	**RAW**	0.196	0.187	0.203	0.179
	**LOG**	0.151	0.146	0.156	0.156
	**PPMI**	0.152	0.144	0.152	0.125
**CBOW**	**RAW**	0.146	0.139	0.150	0.136
	**LOG**	0.131	0.131	0.131	0.145
	**PPMI**	0.127	0.119	0.127	0.113
**GloVe**	**RAW**	0.333	0.343	0.354	0.396
	**LOG**	0.250	0.259	0.255	0.301
	**PPMI**	0.346	0.339	0.350	0.337

**Fig 1:**
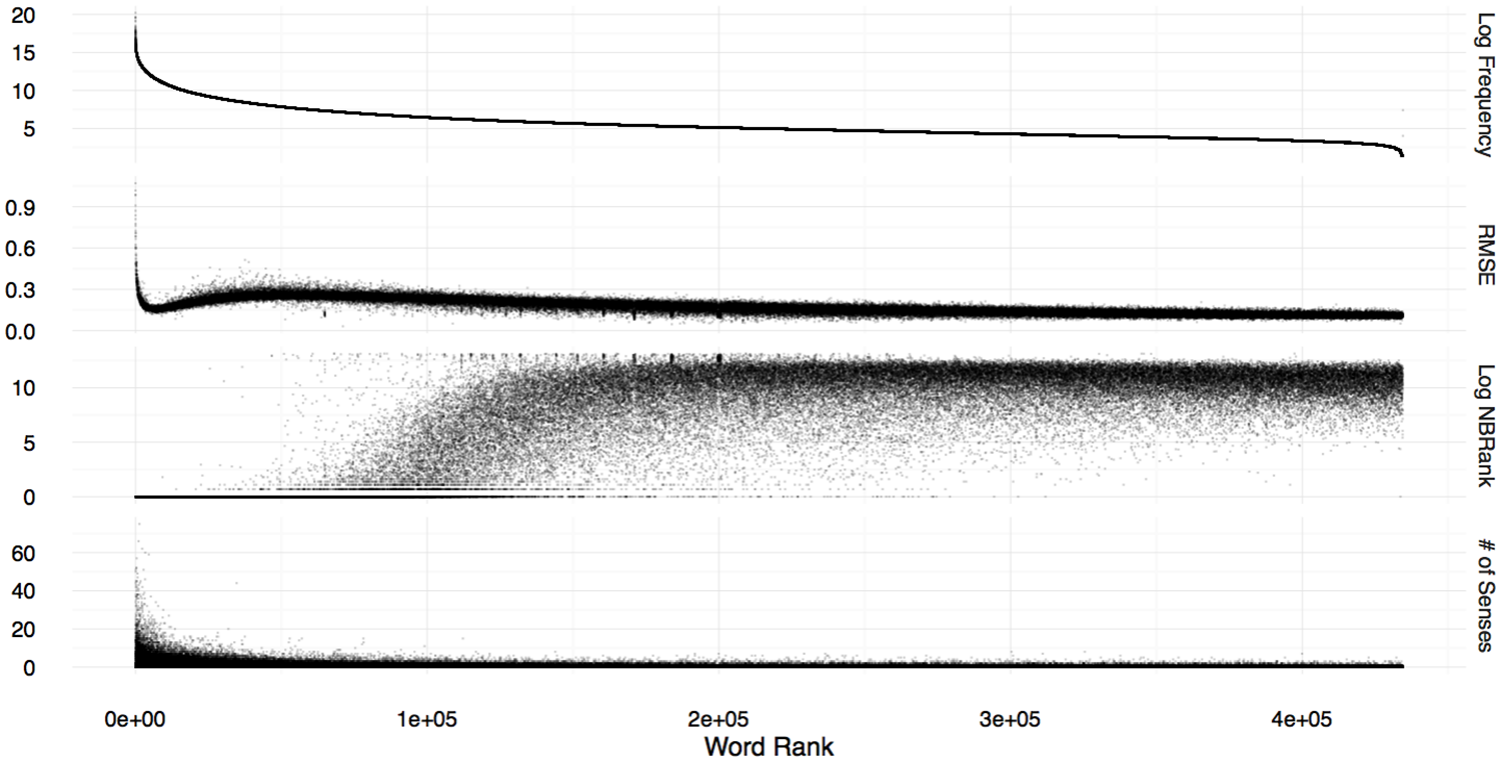
Relationship between word frequency, reconstruction error (train RMSE), rank of the target word in list of nearest neighbours (NBRank) computed using the projected embeddings, and the number of senses according to the WordNet for each word. A linear transformation is learnt between **LOG** source embedding and **GloVe** target embedding using the proposed method.

Fig 1 shows the distributions of (a) the logarithm of the word frequency, (b) reconstruction error (i.e. training RMSE), (c) rank of the target word in the list of nearest neighbours computed using the projected embeddings, and (d) the number of word senses.

For a word w∈V , we compute the cosine similarity between its projected word embedding, ${\hat{w}}^{\left(T\right)}$, and the target word embeddings $u^{\left(T\right)}$ of each word u∈V, and rank *u* in the descending order of the similarity scores. If the target embedding of *w* is ranked higher in this ranked list of nearest neighbours, then we can conclude that the projected embeddings are similar to the actual target embeddings of words. Unlike the RMSE-based evaluation we presented above, the rank of a word in the projected target embedding space considers only the relative position of projected embeddings. Third plot from the top in Fig 1 shows the logarithm of the rank in the nearest neighbour list (log(*NBRank*)) (vertical axis) against the rank of the word according to its frequency in the corpus (horizontal axis). From Fig 1, we see that for high frequent words (ranks up to 10^5^), the proposed method ranks the target word among the top 100 nearest neighbours.

We counted the number of word senses for each word in the WordNet to determine the number of word senses per word. Words that do not appear in the WordNet are ignored in this analysis. In the bottom plot in Fig 1, we show the number of different senses of a word in the vertical axis, whereas the words are ranked by the number of senses they have in the horizontal axis. From Fig 1, we see that except for a small number of common words such as articles and prepositions placed at the first few hundred words in the distribution, our proposed method accurately reconstructs the original **GloVe** embeddings with small reconstruction errors, for a range of words. Interestingly, even for polysemous words for which multi-prototype embeddings [29, 30] must be learnt can nevertheless be linearly transformed using the proposed method. Similar distributions were obtained with **SGNS** and **CBOW** as the target embeddings.

### 4.2 Predicting word similarity and analogy

To evaluate the amount of word semantics preserved during the linear transformation process, we evaluate the predicted target embeddings in two tasks: semantic similarity measurement, and word analogy detection. Both those tasks are frequently used as benchmarks for evaluating word embedding learning methods [31, 32].

For the similarity measurement task we use seven datasets: Rubenstein-Goodenough (**rg**, 65 word-pairs) [33], Miller-Charles (**mc**, 30 word-pairs) [34], rare words dataset (**rw**, 2034 word-pairs) [35], Stanford’s contextual word similarities (**scws**, 2023 word-pairs) [16], SimLex-999 (**simlex**, 999 word-pairs) [36], WordSimilarity-353 dataset (**ws**, 353 word-pairs) [37], and the **men** test collection (3000 word-pairs) [38].

Each word-pair in those datasets has a manually assigned similarity score. We compute the cosine similarity, $\text{cos}\left({\hat{u}}^{\left(T\right)}{\hat{v}}^{\left(T\right)}\right)$, between the predicted target embeddings for the two words *u* and *v* in a word-pair, and use the Spearman correlation coefficient as the evaluation measure to compare predicted similarity scores against the gold standard ratings. Spearman correlation coefficient ranges in [−1, 1], and high values indicate a better agreement of the predicted target embeddings with the human notion of semantic similarity.

**Fig 2:**
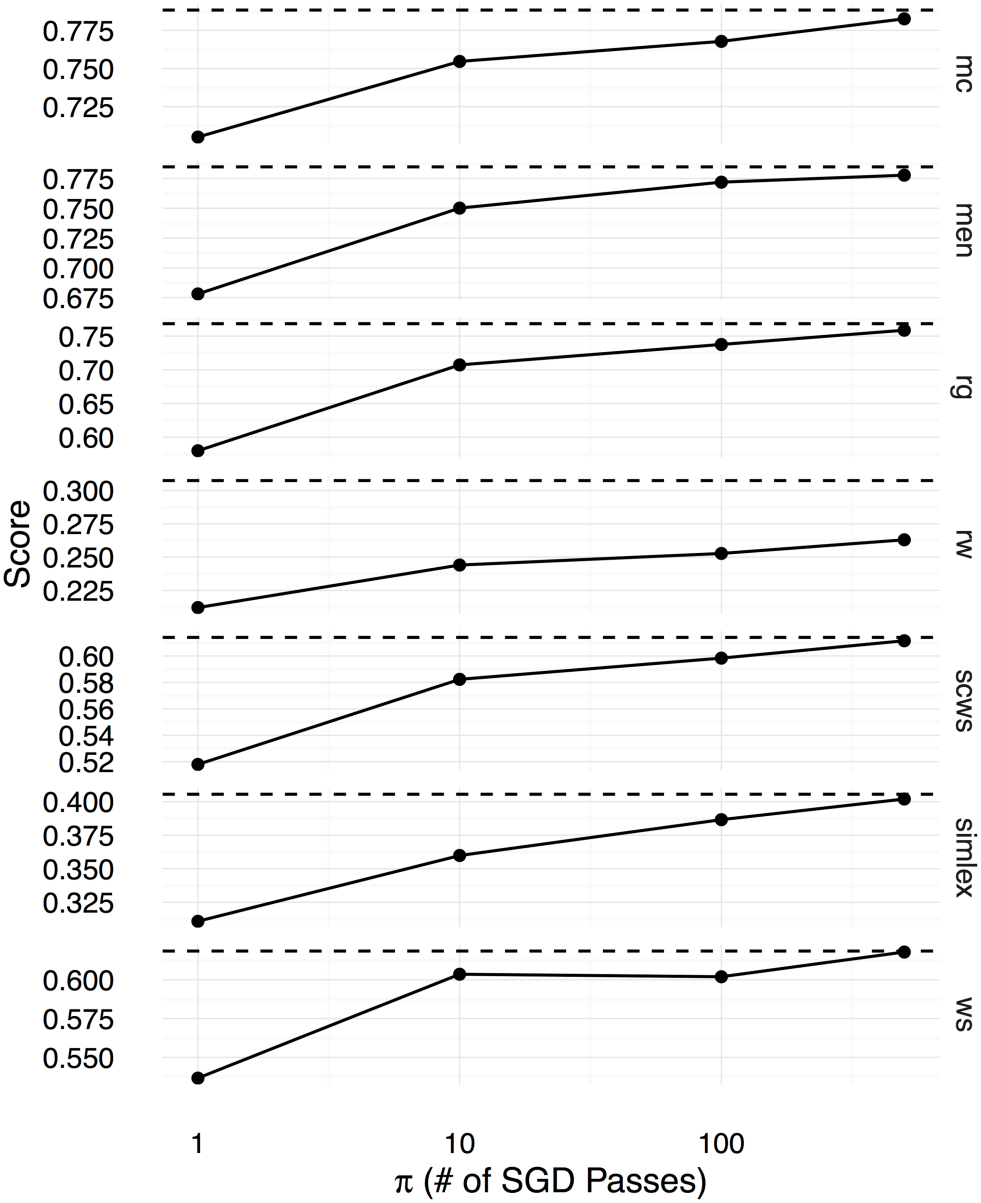
Spearman correlation coefficients (y-axis) between the cosine similarity scores computed using the learnt word embeddings and human ratings in the benchmark datasets are shown for the CBOW embeddings as functions over the number of SGD iterations (x-axis).

**Fig 3:**
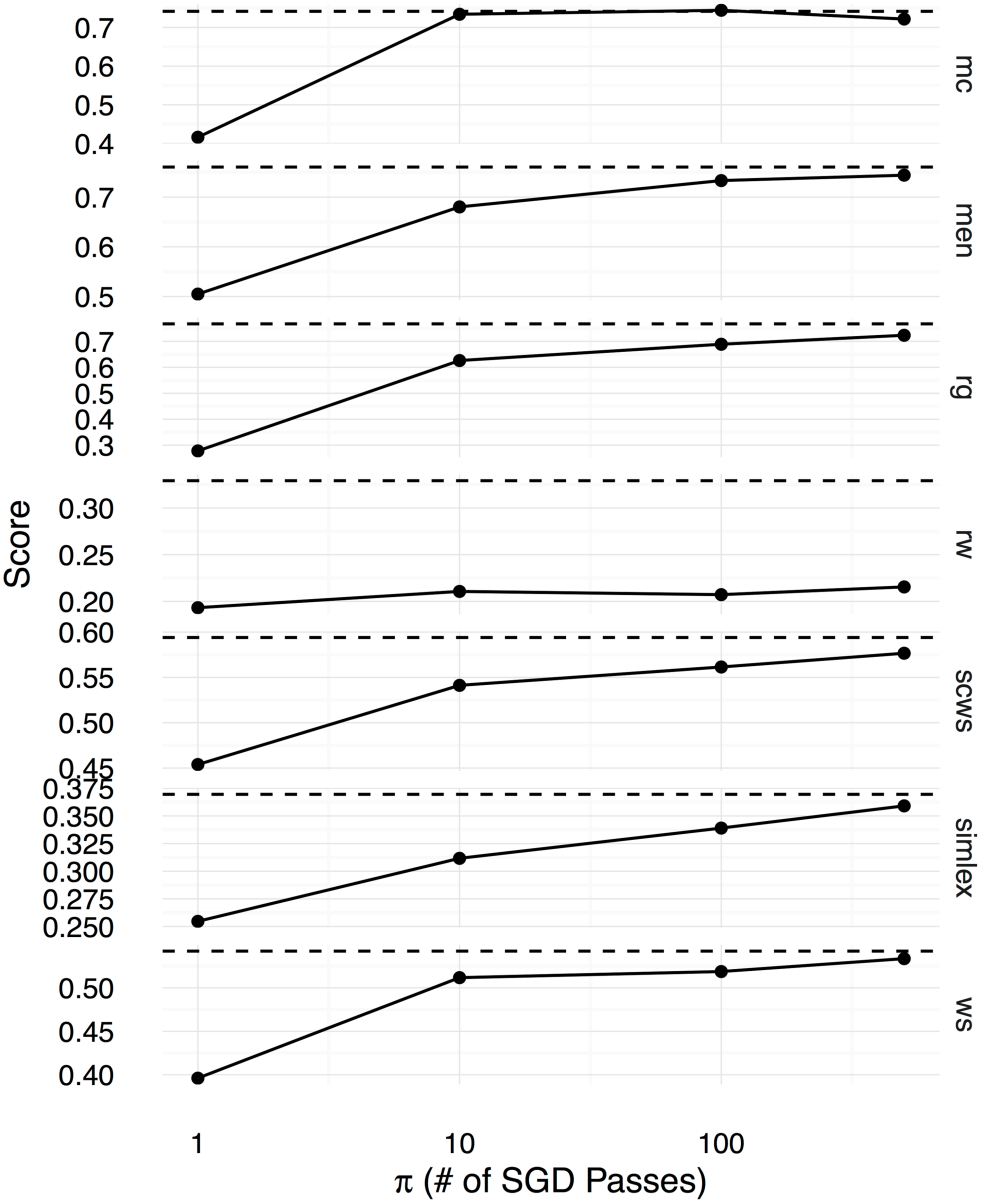
Spearman correlation coefficients (y-axis) between the cosine similarity scores computed using the learnt word embeddings and human ratings in the benchmark datasets are shown for the SG embeddings as functions over the number of SGD iterations (x-axis).

**Fig 4:**
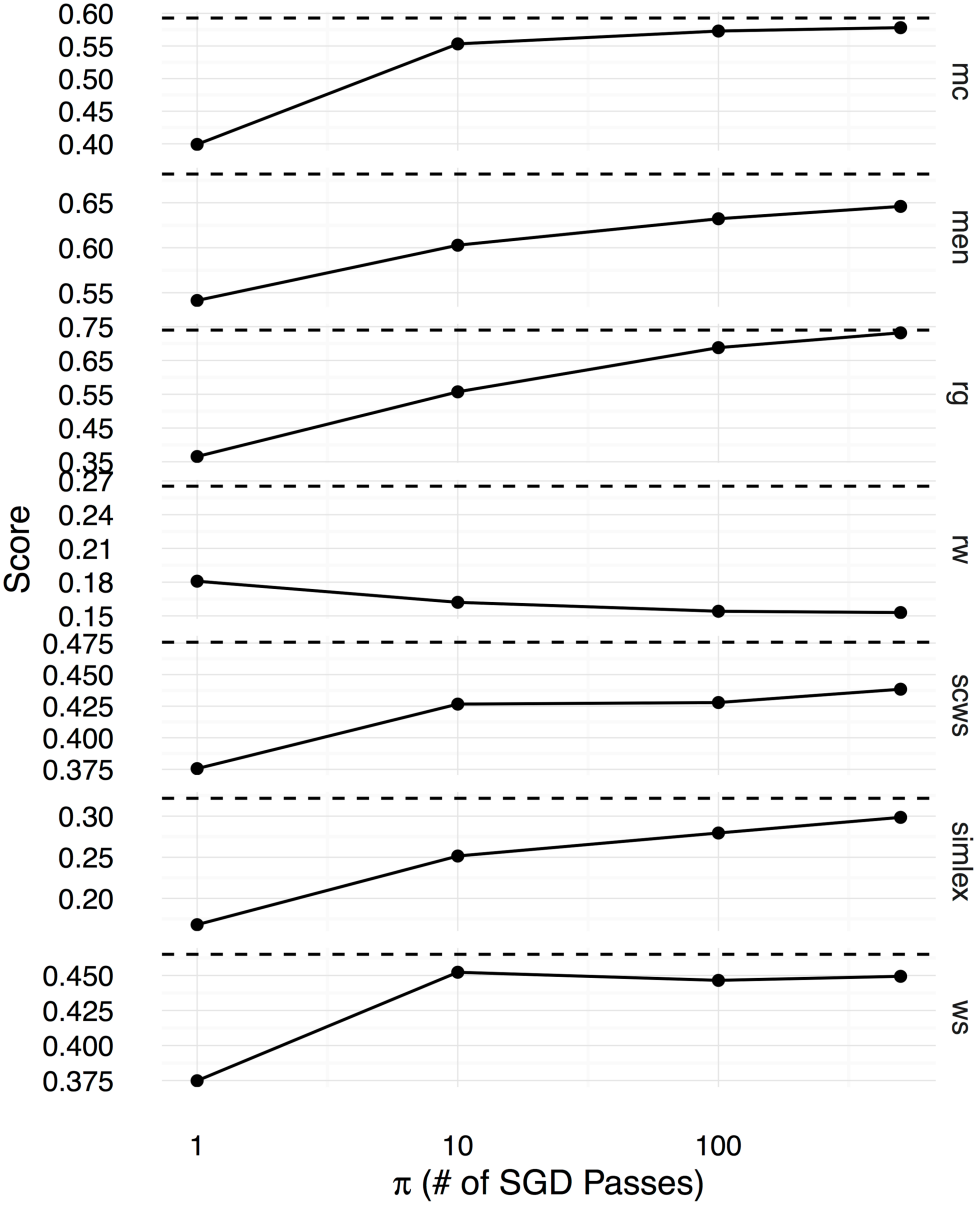
Spearman correlation coefficients (y-axis) between the cosine similarity scores computed using the learnt word embeddings and human ratings in the benchmark datasets are shown for the GloVe embeddings as functions over the number of SGD iterations (x-axis).

**Fig 5:**
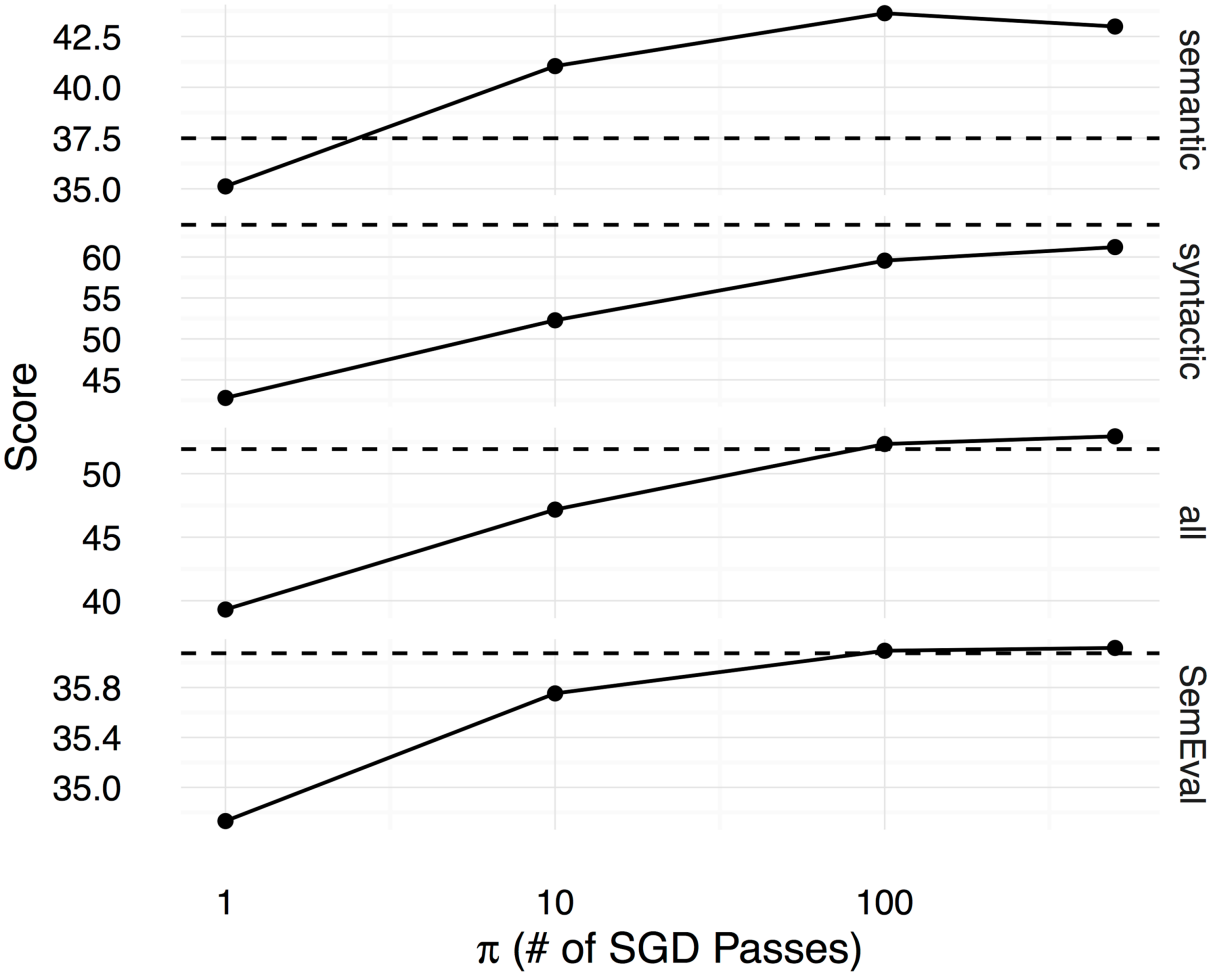
Accuracies (y-axis) for solving word analogy problems on the Google dataset (semantic analogies, syntactic analogies, and all analogies including semantic and syntactic), and max-diff scores on the SemEval dataset are shown for the CBOW embeddings as functions over the number of SGD iterations (x-axis). CosAdd method is used on the Google dataset to predict the correct answer for the word analogy questions.

**Fig 6:**
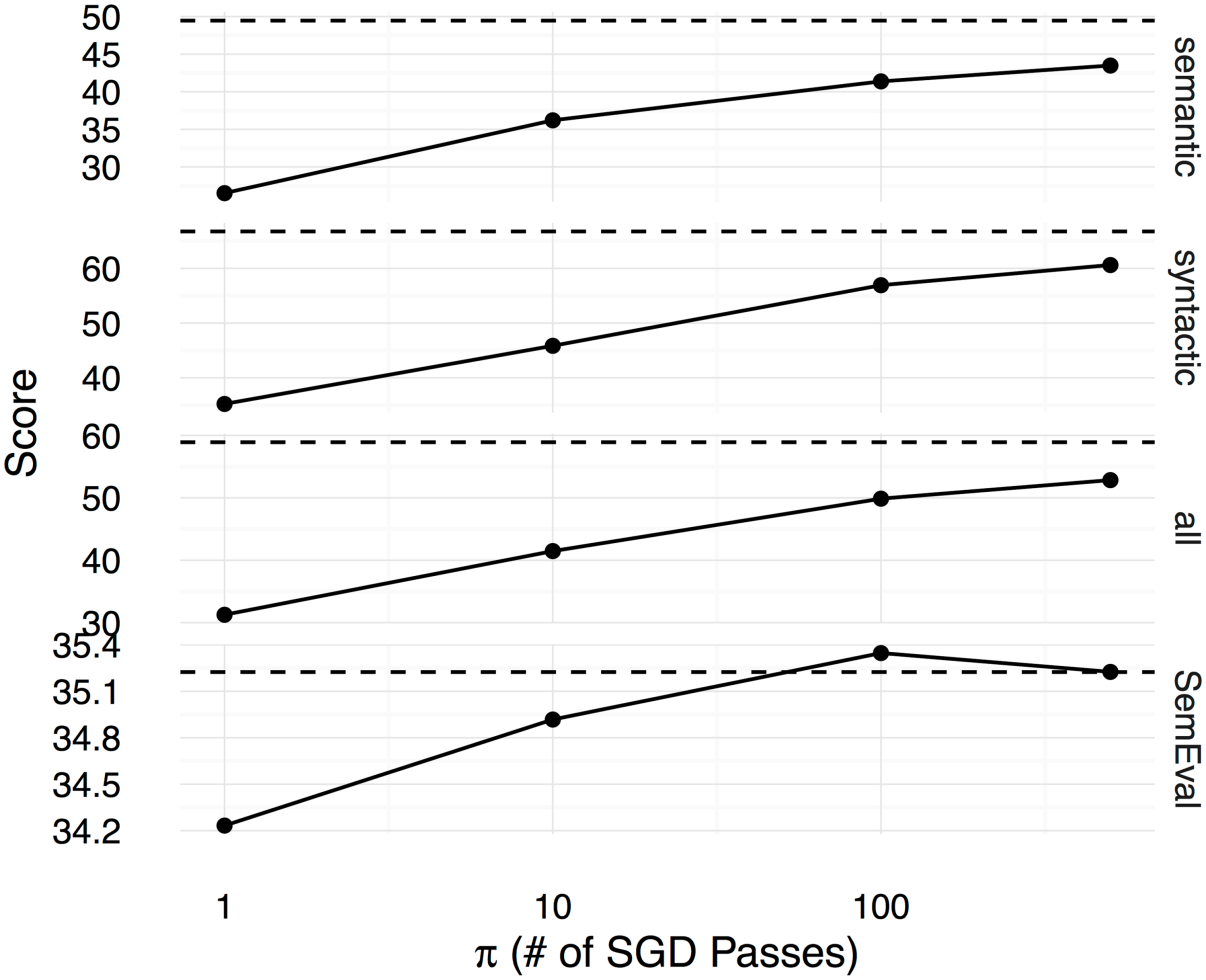
Accuracies (y-axis) for solving word analogy problems on the Google dataset (semantic analogies, syntactic analogies, and all analogies including semantic and syntactic), and max-diff scores on the SemEval dataset are shown for the SG embeddings as functions over the number of SGD iterations (x-axis). CosAdd method is used on the Google dataset to predict the correct answer for the word analogy questions.

**Fig 7:**
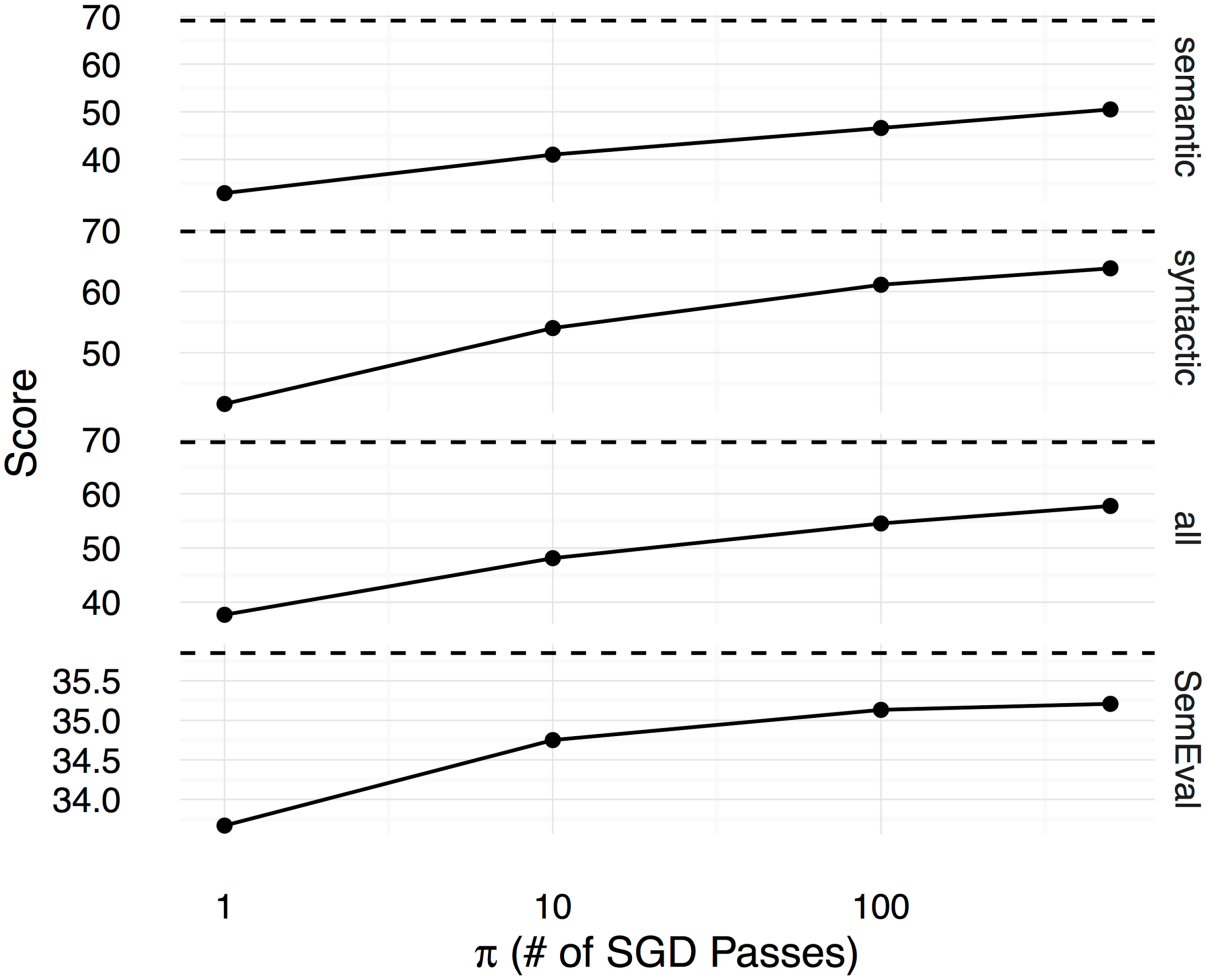
Accuracies (y-axis) for solving word analogy problems on the Google dataset (semantic analogies, syntactic analogies, and all analogies including semantic and syntactic), and max-diff scores on the SemEval dataset are shown for the GloVe embeddings as functions over the number of SGD iterations (x-axis). CosAdd method is used on the Google dataset to predict the correct answer for the word analogy questions.

Figs 2, 3 and 4 show the Spearman correlation coefficients for the similarity predictions made using a linear transformation learnt between the counting-based source embedding **LOG**, and the three prediction-based target embeddings respectively **CBOW**, **SGNS**, and **GloVe**. The level of correlation obtained by the original target embedding is shown as a dashed horizontal line in each subplot, whereas the performance of the linear transformation after *π* number of SGD iterations is shown as a solid line. Overall, we see that the correlation coefficients increase with *π*, reaching the level of the original prediction-based embeddings after 500 iterations. Therefore, with a sufficiently large number of SGD iterations, the proposed method can learn linear transformations that capture almost all the word semantics encoded in the original prediction-based target embeddings. We use the Google word analogy dataset [19], consisting of **semantic** (8869 questions) and **syntactic** (10675 questions) proportional analogies, and the SemEval 2012 Task 2 dataset (**SemEval**, 79 categories) [39] to evaluate the ability to solve word-analogy problems by the proposed linear transformation learning method. In Figs 5, 6, and 7, we report word analogy solving accuracies for the CosMult method that has shown to produce the best results [40] respectively for **CBOW**, **SG**, and **GloVe** embeddings.

Figs 5, 6 and 7 show the scores (percentage of the correctly answered analogy questions on the Google dataset, and the correlation score computed using the official evaluation tool for the SemEval dataset) for the different methods under varying numbers of SGD iterations. Likewise in the semantic similarity experiment, here too we see that with sufficiently large numbers of SGD iterations, the linear transformations learnt using the proposed method can capture the semantics encoded in the original target embeddings. Although for many other tasks and projected embeddings the performance is lower compared to the original target embeddings, surprisingly, in a few tasks (**semantic** and **SemEval** for **CBOW**, and **SemEval** for **GloVe**), the performance is even better than the original embeddings. This tells us that the original embedding is not necessarily optimal, and our proposed method can learn better embeddings in some cases. One possible reason for this improvement could be because of the *ℓ*_2_ regularisation and SGD learning, which prevent overfitting to the original embedding space.

### 4.3 Visualising the learnt projections

Projection matrix **C** projects counting-based source embeddings to prediction-based target embeddings. In counting-based embeddings, each dimension is explicitly annotated with a single word representing some semantic concept, whereas in prediction-based embeddings the semantics of each dimension remain implicit. Therefore, the projection matrix **C** can be thought of as a mapping between these two explicit and implicit embedding spaces. To visualise this relationship, we compute the heatmap for some selected rows of **C**, corresponding to different context words that appear as dimensions in the counting-based source word embeddings. If the mapping between the two embedding spaces is accurately learnt, we would expect the words that belong to the same class to have the same active dimensions.

**Fig 8:**
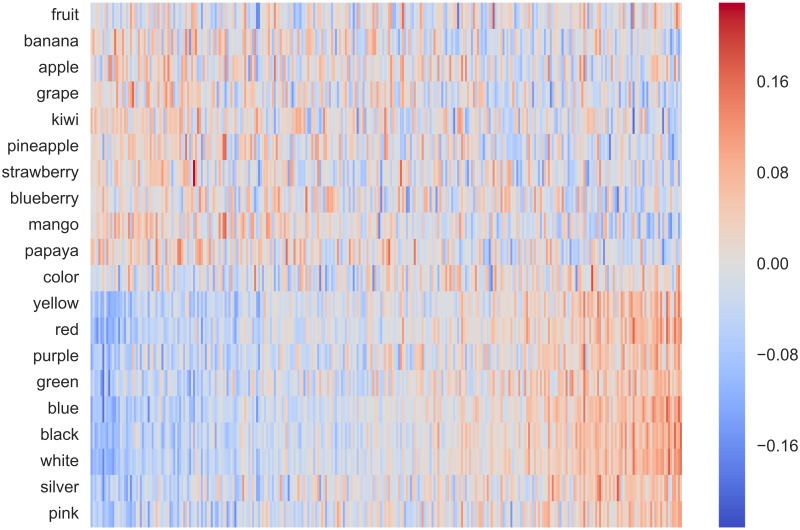
Heatmap for the projections for fruits vs. colours.

**Fig 9:**
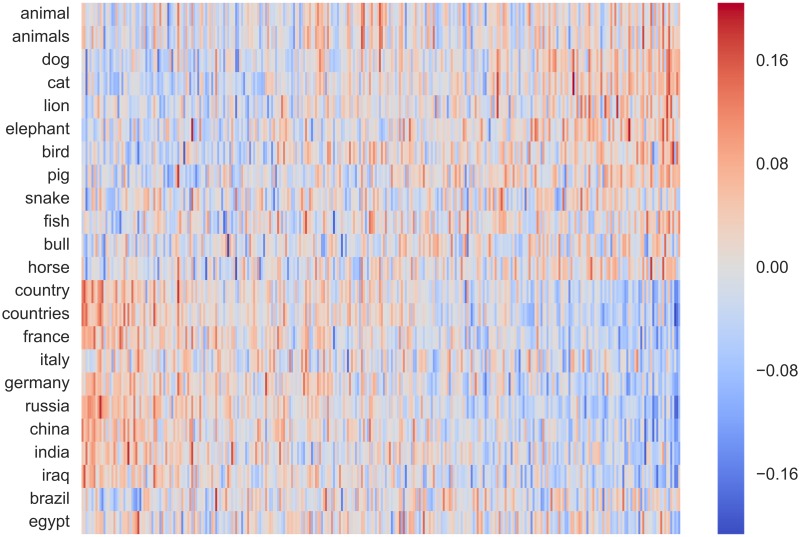
Heatmap for the projections for animals vs. countries.

Following prior work on word representation learning [41, 42], in Figs 8 and 9 show the heatmaps respectively for *fruits* vs. *colours*, and *animals* vs. *countries*. From both figures we see that dimensions of the prediction-based embeddings (shown in the horizontal axis) that correspond to the dimensions of the counting-based embeddings (shown in the vertical axis) are different for each context word compared. Note that the ordering of dimensions in each axis is arbitrary and we have used different permutations in each figure to emphasise the association.

The heatmaps shown in Figs 8 and 9 can be considered as an *interpretation* of the dimensions in the counting-based embedding in terms of the dimensions in the prediction-based embedding. We see from the two figures that the same latent dimension in the prediction-based embedding is associated with multiple dimensions in the counting-based embedding. Considering that the dimensionality of the prediction-based embedding (ca. 300) is much smaller than that of the counting-based embedding (ca. 434k), it is natural that a single latent dimension must encode a broader class of semantics represented by multiple dimensions in the counting-based embedding if the two embeddings to capture the same information. Simply ranking dimensions in the counting-based embedding by the values of the elements in **C** is inadequate to obtain meaningful associations because elements in **C** can be both positive as well as negative. More advance alignment techniques such as bipartite graph-matching using max-flow methods could be useful here.

## 5 Conclusions

We proposed a method to learn linear transformations between several counting-based and prediction-based word embeddings. Our proposed method does not depend on the underlining word embedding learning algorithm, hence applicable when finding a linear transformation between any two pre-trained word embeddings. This property is particularly attractive because the proposed method can be used as a post-processing analysis tool for aligning the dimensions between different word embeddings.

We specifically considered the scenario where we would like to find a linear transformation between prediction-based word embeddings where the dimensions are implicit and randomly initialised, and counting-based word embeddings where the dimensions are explicitly annotated with words. This mapping is useful for providing an interpretation for the implicit dimensions in prediction-based word embeddings using the explicit dimensions in the counting-based word embeddings. It shows that each context word in the counting-based embedding can be associated with a subset of the dimensions in the prediction-based embedding.

Our experimental results show that counting-based embeddings of most words can be linearly projected to the vector space spanned by the prediction-based word embeddings. This result is important given that the two types of word embeddings have shown different performances in different tasks, and prior work analysing those differences [1, 10] have hinted at the close relationship between the two types of embeddings. Moreover, experimental results on similarity and analogy benchmarks show that most of the semantic information in the target embeddings can be captured by the proposed method. Visualisations of the projections learnt by the proposed method for different word classes show that indeed the learnt projections demonstrate a high-level of structure organised by the prototypical semantics represented by those word classes.

Our work open up several interesting future research directions to the NLP community.

- Although linear transformations are simple to interpret and efficient to compute over large vocabularies, it is by no means the only possible transformation between two word embedding spaces. Nonlinear transformations can capture richer relationships between vector spaces as demonstrated repeatedly by the recent successes of deep neural networks. A natural next step would be to explore the possibilities of learning a nonlinear transformation between counting-based and prediction-based word embedding spaces.
- The linear transformation we have learnt using our proposed method is a global transformation that applies to all word embeddings equally. However, as evident by the numerous uses of distributional hypothesis that postulates the meaning of a word can be estimated simply by looking at its nearest neighbours, there is a high degree of locality in natural language semantic spaces. Considering this observation, another research direction would be to learn locally-linear transformations [43] between word embedding spaces.
- Our analysis shows the existence of a linear transformation at macro-level across different classes of words such as by frequency, level of ambiguity, and part-of-speech. However, it remains an interesting open question as to what specific words can be linearly transformed and to what extent. Such a micro-level analysis would reveal further insights into the relationships between counting-based and prediction-based embeddings.
- Eq (1) can be extended to incorporate multiple target embeddings by adding loss terms corresponding to the projection between each target embedding and the single source embedding. The transformation matrix **C** can then be shared across the different loss terms such that we learn a single consistent linear transformation from the source embedding to all of the target embeddings.
- Our proposed method can be used to find a liner transformation between *any* two embeddings, not limited to a counting-based source embedding and a prediction-based target embedding. This is useful to quantitatively understand how one set of embeddings is related to another set of embeddings created using different embedding learning methods, different resources, or different random initialisations of the same algorithm. Two embedding learning algorithms might appear to be different in the objectives that they optimise for and/or the optimisation techniques that they use. However, when trained on the same resources, they might produce similar embeddings, different only by a linear transformation. Our proposed method can be used as a tool to further investigate the embeddings learnt by different embedding learning algorithms. As a special case of such an analysis, the coefficients of the projection matrix provides an interpretation for the alignment between dimensions in the source and target embeddings. The visualisations shown in the paper are a first attempt at such an analysis. We plan to peruse those research directions in our future work.
